# One-Dimensional Motion Representation for Standing/Sitting and Their Transitions

**DOI:** 10.3390/s24216967

**Published:** 2024-10-30

**Authors:** Geunho Lee, Yusuke Hayakawa, Takuya Watanabe, Chunhe Li

**Affiliations:** Graduate School of Engineering, University of Miyazaki, 1-1 Gakuen-Kibanadai Nishi, Miyazaki 8892192, Japan; hi19034@student.miyazaki-u.ac.jp (Y.H.); hi20050@student.miyazaki-u.ac.jp (T.W.)

**Keywords:** standing-and-sitting motions, one-dimensional representation, interface, distance measurements, proximity sensor

## Abstract

In everyday life, people often stand up and sit down. Unlike young, able-bodied individuals, older adults and those with disabilities usually stand up or sit down slowly, often pausing during the transition. It is crucial to design interfaces that accommodate these movements. Additionally, in public settings, protecting personal information is essential. Addressing these considerations, this paper presents a distance-based representation scheme for the motions of standing up and sitting down. This proposed scheme identifies both standing and sitting positions, as well as the transition process between these two states. Our scheme is based solely on the variations in distance between a sensor and the surfaces of the human body during these movements. Specifically, the proposed solution relies on distance as input, allowing for the use of a proximity sensor without the need for cameras or additional wearable sensor attachments. A single microcontroller is adequate for this purpose. Our contribution highlights that using a proximity sensor broadens the applicability of the approach while ensuring that personal information remains secure. Additionally, the scheme alleviates users’ mental burden, particularly regarding privacy concerns. Extensive experiments were performed on 58 subjects, including 19 people over the age of 70, to verify the effectiveness of the proposed solution, and the results are described in detail.

## 1. Introduction

Standing up and sitting down are seemingly simple actions that most people repeat numerous times each day. However, elderly individuals and those with muscle diseases or disorders may find these movements challenging. The ability to stand up from a seated position and return is crucial for maintaining independence in daily living. Various assistive technologies [1,2,3,4] have been developed to assist with these motions. Among these, recognition methods for standing up and sitting down have been extensively researched. These approaches are used to monitor the health of elderly individuals in nursing homes [5,6,7,8], diagnose lower-limb diseases and assess locomotion in hospitals [9,10,11,12], and develop interfaces for assistive systems [13,14]. Measurement methods serve as the core technology for these approaches. Based on their sensor installation, measurement tools are primarily categorized into two types: wearable and nonwearable devices.

Wearable devices for measuring human activities are increasingly being reported. These devices incorporate sensors such as inertial measurement units (IMUs), barometers, pressure sensors, and electromyography (EMG) sensors. An IMU-based wearable device [10,15,16,17,18,19,20] measures the acceleration and angular velocity of the specific body part to which it is attached. Multiple sensors must be integrated into the device to assess the posture of the entire body. A wearable barometer [21,22,23] provides the height of the part of the body’s position, whether standing or sitting, to which it is affixed. Since this wearable device is influenced by environmental conditions, the sensor values must be calibrated frequently. Next, wearable devices equipped with pressure sensors [1,24,25,26] are used to detect variations in forces on the contacted surfaces. However, these pressure sensors are sensitive to temperature and encounter challenges owing to signal acquisition noise. Additionally, EMG sensor-type wearable devices [8,9,27,28,29] use muscle potential and frequency analysis to identify changes in fatigue resulting from muscle movements. However, inadequate skin contact hampers stable signal acquisition. These wearable devices facilitate the measurement and swift recognition of standing-up and sitting-down motions, thus eliminating the need to monitor computer screens. Most of the devices mentioned are portable, hands-free tools; however, they exhibit a notably short battery life. Additionally, their size and the effort needed to wear them render them impractical for daily use. A drawback of several wearable devices is the requirement for wires connecting the sensors to their electronic terminals. While these wires are essential for transmitting signals from the human body, they further impede the practicality of these devices for everyday tasks.

Several studies have detailed the measurement methods for typical nonwearable devices. First, these devices use an interface scheme that relies on visual input from a camera [12,30,31,32,33,34]; this visual input can capture three-dimensional posture data of the entire body from a single point or frame. By improving the identification of related motion features, these visual data enable the interface scheme to detect emerging trends. In recognizing standing and sitting motions, camera-based interface schemes efficiently and quickly convey motion intents, thereby expediting the decision-making process. However, the quality of their data is influenced by lighting conditions and environmental factors. Additionally, the expenses associated with processing large amounts of information must be taken into account. Finally, privacy protection is a critical consideration when camera systems are used in sensitive areas such as toilets or bathrooms. Second, a force plate [12,34,35,36,37], which consists of multiple force sensors, measures the ground reaction force exerted on the human body during standing-up or sitting-down movements. The input from the force plate can be used to analyze the movement of the center of mass. In addition, by detecting the ground reaction force, the force plate enables the measurement of postural stability, explosive force, and leg strength and power. However, the maintenance and calibration of a force plate are expensive.

Based on our survey, we summarize the interface requirements for standing and sitting motions here. First, physical strength diminishes with age, resulting in slower movements and reflexes among the elderly. Second, older individuals often find complex controls with small characters and intricate buttons challenging to learn and use. Third, because these interfaces are expected to be used in diverse locations, protecting users’ personal information is essential. In public spaces, it is important to implement noncontact maneuvering to the greatest extent possible in the post-COVID-19 era.

In light of the aforementioned considerations, this study addresses the interface issue related to representing the motions of standing up and sitting down. Our proposed solution involves using distance measurements from a fixed sensor to the body of an individual in either a standing or sitting position. The underlying concept is as follows. When observed from the side, a standing body appears as a straight line, while the sitting body forms a bent line at the waist and knee. Based on this idea, we propose a distance-based representation scheme for the standing position, the sitting position, and the transition between these two positions. The core technology behind this solution involves the conversion of one-dimensional (1D) information (distance) and the estimation of lower-limb joints, leading to the creation of a linkage model consisting of three rotational joints. A notable advantage of this solution is its reliance on 1D distances, allowing it to function with a proximity sensor that only requires a single microcontroller, eliminating the need for additional memory. This representation scheme broadens the application potential of the solution beyond spatial limitations. Given its applications, such as with public transport straps, these interfaces should be compact and portable, and it is also preferable to maintain low computational costs for recognition processes. For example, each strap in a public transport vehicle should ascend and descend as its user (passenger) stands up and sits down, respectively.

This paper is structured as follows. Section 2 outlines our research problem and its definitions. Section 3 provides an explanation of the proposed solution. Section 4 details the results from extensive experiments performed to evaluate the effectiveness and performance of the proposed approach, along with a discussion of these findings and potential future directions. Finally, Section 5 presents our conclusions.

## 2. Problem Statement

### 2.1. Problem Definition

**Figure 1:**
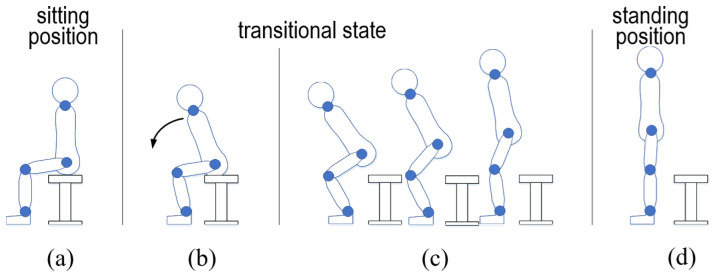
Illustration of the standing-up motion process from sitting position to standing position ((**a**) preparation, (**b**) standing phase I, (**c**) standing phase II, (**d**) standing phase III).

**Figure 2:**
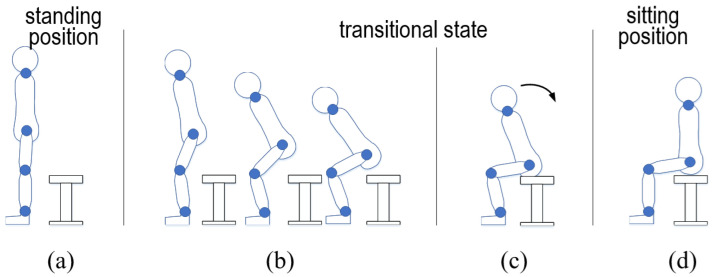
Illustration of the sitting-down motion process from standing position to sitting position ((**a**) preparation, (**b**) sitting phase I, (**c**) sitting phase II, (**d**) sitting phase III).

We aim to create an interface model for the standing-up and sitting-down motions without requiring additional sensor equipment or compromising privacy. Typically, these motions are categorized into two primary patterns: standing and sitting positions. The distinction between these positions can be determined by measuring the difference in distance from the sensor to the surfaces of the human body in each position. However, additional effort is essential to achieve a more sophisticated representation of the standing-up and sitting-down motions. First, as illustrated in Figure 1 and Figure 2, the transitional states between the sitting and standing positions must be clearly defined. Elderly individuals tend to raise or lower themselves slowly and often pause during this transition. Second, aligning with the aforementioned requirement, the transition process should be monitored in sync with human motions for the effective use of assistive systems. Third, experiments involving participants across a broad age range should be performed to validate the effectiveness of the proposed approach.

To meet these requirements, we study the representation of standing-up and sitting-down motions. By connecting the physical characteristics of the elderly to these motion processes, it becomes essential to describe the transitional states for a clear estimation of these motions. Consequently, we pose the following problem: *How can the standing-up and sitting-down motions, along with their individual transition processes, be described and modeled using distances?*

This problem is considered a representation challenge between human motions and a sensor system. Our approach uses a distance measurement-based model, which facilitates the representation of standing and sitting positions as well as the transition process between them. These motions replace a linkage model to streamline the representation of the human body. Based on this linkage model, we propose a distance-based representation scheme, which is detailed in the following subsection.

### 2.2. Definitions of Measurement Model

**Figure 3:**
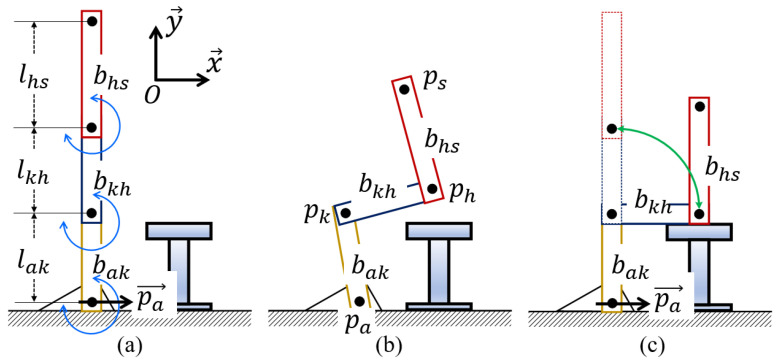
Definitions and notations of linkage representation where the linkage is composed of three rigid bars and three joints ((**a**) standing position, (**b**) transition process, and (**c**) sitting position).

To start, we introduce the model definition for the standing-up and sitting-down motions. A two-dimensional (2D) observation plane is designated as the sagittal plane. As shown in Figure 3, the horizontal axis parallel to the ground is defined as $\vec{x}$, while the axis that is counterclockwise and perpendicular to $\vec{x}$ is denoted by $\vec{y}$. The intersection point of $\vec{x}$ and $\vec{y}$ is labeled as *O*. Following this, we present three types of lengths measured from the ground along $\vec{y}$. First, $l_{c}$ represents the height of the stool. Second, for an individual seated on a stool of $l_{c}$, $l_{k}$ represents the distance from the ground to the knee $p_{k}$, with the assumption that $l_{k}$ is greater than $l_{c}$. Third, for a person in a standing posture, the height from the ground to a section of the thigh is denoted by $l_{se}$, which is greater than $l_{k}$. Lastly, $p_{se}$ refers to a point that is positioned at a height of $l_{se}$ from the ground along $\vec{y}$.

The linkage model, consisting of three rigid bars, is created to simplify the motions of standing up and sitting down. In the linkage model shown in Figure 3b, $p_{a}$, $p_{k}$, $p_{h}$, and $p_{s}$ denote the ankle, knee, hip, and shoulder joints, respectively. From bottom to top, the three bars are as follows: the bar connecting the foot to the knee ($b_{ak}$), the bar connecting the knee to the pelvis ($b_{kh}$), and the bar connecting the pelvis to the shoulder ($b_{hs}$). Next, several geometric definitions are presented. $\bar{p_{a}p_{k}}$, $\bar{p_{k}p_{h}}$, and $\bar{p_{h}p_{s}}$ denote the straight lines connecting $p_{a}$ to $p_{k}$, $p_{k}$ to $p_{h}$, and $p_{h}$ to $p_{s}$, respectively. The lengths between individual joints, from bottom to top, are defined as $l_{ak}$, $l_{kh}$, and $l_{hs}$. In addition, ${\vec{p}}_{a}$ denotes a vector that is parallel to $\vec{x}$ and passes through $p_{a}$. Using these straight lines, the angles formed between ${\vec{p}}_{a}$ and $\bar{p_{a}p_{k}}$, between $\bar{p_{a}p_{k}}$ and $\bar{p_{k}p_{h}}$, and between $\bar{p_{k}p_{h}}$ and $\bar{p_{h}p_{s}}$ are defined as $\theta_{a}$, $\theta_{k}$, and $\theta_{h}$, respectively.

Moreover, $b_{ak}$, $b_{kh}$, and $b_{hs}$ are assumed to be rotatable at $p_{a}$, $p_{k}$, and $p_{h}$ to illustrate the motions of standing up and sitting down. In this linkage, these motions are assumed to occur solely in the anteroposterior (front to back) and vertical (top to bottom) directions. In the standing position, $b_{ak}$, $b_{kh}$, and $b_{hs}$ are aligned along the same line (Figure 3a). In the sitting position (Figure 3c), $b_{ak}$, $b_{kh}$, and $b_{hs}$ are oriented at right angles to each other. There are also transitional states observed between the standing and sitting positions (Figure 3b).

## 3. Distance-Based Representation Scheme

### 3.1. Measurements Using Proximity Sensor

**Figure 4:**
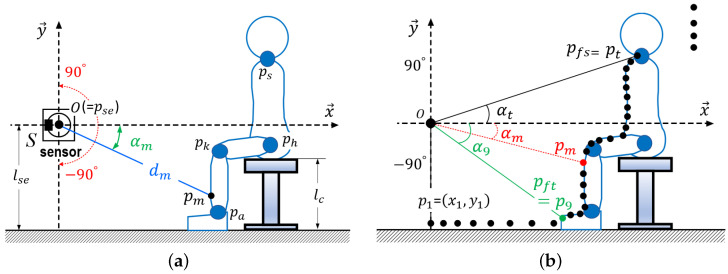
Illustration of measurement method using proximity sensor (*S*) positioned at $p_{se}\;\left(=O\right)$. (**a**) Location of a sensor and measurement parameters; (**b**) Computations by pairs of distances and angles.

We outline the measurement method for the standing-up and sitting-down motions. As shown in Figure 4a, the sensor (*S*) is located at $p_{se}$ with the center of *S* defined as *O*$\left(=p_{se}\right)$. Scanning is performed by *S* across a range of $-90^{{}^{\circ}}$ to $+90^{{}^{\circ}}$ at predetermined intervals. The rotation time of *S* is designated as one sampling time *t*. The input number for *t* is represented as 1,⋯,m,⋯,ss. The distance from $p_{se}$ to the body surface is denoted as $d_{m}$, and the scanning angle of *S* corresponding to $d_{m}$ is defined as $\alpha_{m}$. Using trigonometric functions with $d_{m}$ and $\alpha_{m}$, a point ($p_{m}=\left(x_{m},y_{m}\right)$) is determined. After one rotation of *S*, as depicted in Figure 4b, a set of $p_{m}$ is collected. It is assumed that the feet do not move along the $\vec{x}$ direction while a person is standing up or sitting down. Additionally, in both standing and sitting positions, $b_{ak}$, $b_{kh}$, and $b_{hs}$ remain stationary, with no surging or swaying occurring.

### 3.2. Overall Computation Flow

**Figure 5:**
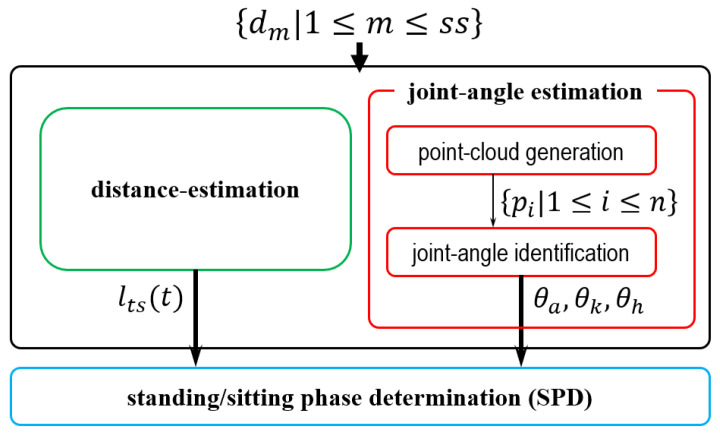
Computation flow of the distance-based representation scheme, with detailed explanations of individual modules provided in the subsequent subsections.

Figure 5 shows the computation flow of the proposed distance-based representation scheme. The input consists of $\left\{d_{m}|1\leq m\leq ss\right\}$, derived from *S*, while the output denotes phase determination (either standing or sitting). Using $d_{m}$, the distance estimation and joint angle estimation modules calculate the distance changes from the standing position to the sitting position, as well as the joint angles $\theta_{a}$, $\theta_{k}$, and $\theta_{h}$, respectively. These modules operate in parallel. At time *t*, the motion phase can be identified using the outputs from the standing/sitting phase determination (SPD) module. Detailed descriptions of these modules and their computation flows are provided in the subsequent subsections. In summary, at *t*, the representation scheme calculates the motion process based on $d_{m}$, producing the determination result as the current phase. Notably, the scheme performs scheduling at *t* recursively without retaining any prior data.

### 3.3. Distance Estimation Module

**Figure 6:**
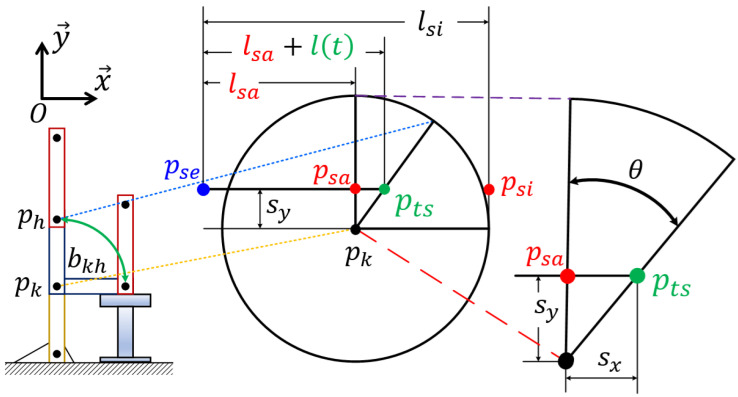
Illustration of l(t) computation based on the rotation of $b_{hk}$ centered at $p_{k}$ and $l_{ts}$ formula.

The distance estimation module is designed to identify standing and sitting positions by calculating the distance change l(t) during the actions of standing up and sitting down. This module computes and outputs l(t) based on *t*. The computation process of l(t) is illustrated in Figure 6.

First, three types of distances are defined. For the sitting position, $l_{si}$ represents the distance from $p_{se}$ to a point $p_{si}$ on the body surface. In the standing position, the distance from $p_{se}$ to a point $p_{sa}$ on the body surface is expressed as $l_{sa}$. During the standing-up and sitting-down motions, a predetermined beam of *S* identifies a point $p_{ts}$ on the body surface, where $l_{ts}$ denotes the distance from $p_{se}$ to $p_{ts}$. In the standing position, $l_{sa}$ is equal to $l_{ts}$. In the sitting position, $l_{si}$ and $l_{ts}$ are equivalent. Relative to $p_{se}$, $l_{si}$ can be decomposed into $l_{sa}$ and l(t). This means that, during the motions, $l_{ts}\left(t\right)$ can be expressed as $l_{sa}$ and l(t). Next, to derive l(t), the rotational movement of $b_{kh}$ is examined. Following the conditions and assumptions regarding $b_{kh}$ as detailed in Section 2.2, the standing-up and sitting-down motions on the $\vec{x}\vec{y}$ plane are modeled by the rotation of $b_{kh}$ around $p_{k}$.

The circular movements during these actions create a fan-like shape with an angle of θ$\left(0^{{}^{\circ}}\leq \theta \leq 90^{{}^{\circ}}\right)$, configured by $\bar{p_{k}p_{sa}}$ and $\bar{p_{k}p_{ts}}$. In other words, the changes in this fan shape can be seen as the reciprocating motions between $p_{sa}$ and $p_{si}$. Since these reciprocating motions can be represented by the distance from $p_{se}$ to $p_{ts}$, the standing and sitting positions can be tracked by measuring the variation in this distance.

With reference to $p_{k}$, two types of distances with respect to $\vec{x}$ and $\vec{y}$ are illustrated in Figure 6. First, the distance between $p_{k}$ and $p_{sa}$ (or $p_{ts}$) in the $\vec{x}$ direction is defined as $S_{x}$. Second, $S_{y}$ represents the distance between $p_{k}$ and $p_{sa}$ in the $\vec{y}$ direction. When $S_{x}=0$, the distance from $p_{se}$ to $p_{sa}$ is denoted as $l_{sa}$, indicating the standing position. According to the definition of θ, if $S_{x}$ is nonzero, it is determined by equation $S_{x}=S_{y}\tan\theta$. Therefore, from the aforementioned geometric considerations, the distance $l_{ts}$ from $p_{se}$ to $p_{ts}$ is expressed by combining $l_{sa}$ and $S_{x}$, formulated as $l_{ts}=l_{sa}+S_{x}$. Concerning $l_{ts}\left(t\right)$, the variations in $S_{x}$ as a function of *t* are designated as l(t) during the standing-up and sitting-down motions. As a result, the rotational motion can be perceived as a 1D reciprocating motion of l(t) transitioning from $p_{sa}$ to $p_{si}$. From the perspective of circular movement, the angular velocity ω relative to *t* is taken into account. Using the relation ω·t=θ, θ can be calculated.

**Figure 7:**
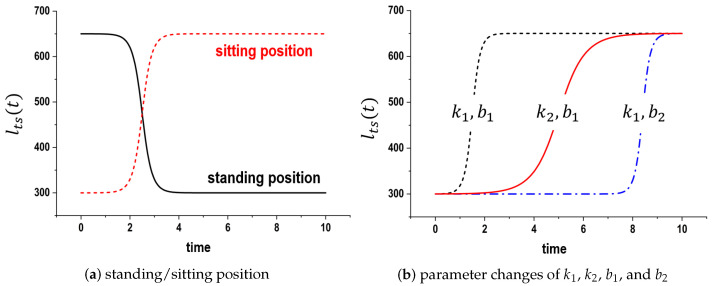
Computational results for $l_{ts}\left(t\right)$ trajectories in the distance estimation module when $l_{sa}=300$ and $l_{si}=650$.

Next, the standing-up and sitting-down motions are analyzed to estimate θ at *t*. Our observations reveal that these motions gradually accelerate at the beginning and then gradually decelerate toward the end. Therefore, the ω value of the fan-shaped motion can be represented as an exponential function. The approximation of this exponential motion is related to the use of the well-known Gudermannian function [38]. Using this function, the rotation is transformed into linear motion as follows:   

(1) $$l_{ts}\left(t\right)=\left\{\begin{array}{cccc} \frac{2}{\pi }\left(l_{si}-l_{sa}\right)\tan^{-1}\left(e^{-k_{i}t+b_{i}}\right)+l_{sa} & \; & & \left(\mathrm{standing}\right)\\ \; & \; & & \\ \frac{2}{\pi }\left(l_{si}-l_{sa}\right)\tan^{-1}\left(e^{k_{i}t-b_{i}}\right)+l_{sa} & \; & & \left(\mathrm{sitting}\right) \end{array}\right.$$

where $k_{i}$ and $b_{i}$ are positive constants, representing the motion speed and starting time parameters, respectively. Based on this equation, Figure 7a shows the trajectories represented by $l_{ts}\left(t\right)$. Individual motions are plotted according to the sign of $e^{k_{i}t}$. The slopes and starting times are modified by adjusting $k_{i}$ and $b_{i}$ as shown in Figure 7b.

### 3.4. Joint Angle Estimation Module

To compute $\theta_{a}$, $\theta_{k}$, and $\theta_{h}$, as detailed in Section 3.2, the joint angle estimation module performs two functions: point cloud generation and joint angle identification. The computation process of this module is as follows.

As illustrated in Figure 4b, the point cloud generation function produces a set of points that represent the body surface. At *t*, $p_{m}$ is calculated using $d_{m}$ and $\alpha_{m}$. Once $p_{1}$, $p_{2}$, ⋯, $p_{m}$, ⋯, $p_{ss}$ are calculated, the points are analyzed to identify the valid range that represents the body surface. Starting from the point $p_{1}$$\left(=\left(x_{1},y_{1}\right)\right)$, which corresponds to the $-90^{{}^{\circ}}$ direction of *S*, the $\vec{y}$ coordinate of each point is checked to see if it exceeds the value of $y_{1}$. If the ordinate is greater, then that coordinate is considered to be the front of the toes. Specifically, this point is designated as $p_{ft}$. After establishing $p_{ft}$, $p_{a}$ is positioned at a predetermined distance $l_{ax}$ along $+\vec{x}$. Subsequently, the distance $dist(p_{m},p_{m+1})$ between $p_{m}$ and $p_{m+1}$ in the direction of $90^{{}^{\circ}}$ of *S* is calculated from $p_{ft}$ to $p_{ss}$. This calculation routine verifies whether $dist(p_{m},p_{m+1})$ exceeds the assigned length $l_{sout}$, which serves as our empirical value. If $dist(p_{m},p_{m+1})$ is greater than $l_{sout}$, then points following the (m+1)*th* are disregarded as part of the body surface. In this context, the *mth* point is defined as $p_{fs}$. This function outputs a set of points that represents the valid range from $p_{ft}$ to $p_{fs}$. For convenience, this set of these points is represented by $\left\{p_{i}|1\leq i\leq n\right\}$, and $p_{fs}$ is empirically considered as $p_{s}$.

Next, the joint angle identification function aims to determine $p_{k}$ and $p_{h}$. The underlying principle of this computation is to locate points with larger $\beta_{i}$ (=$∠p_{i-1}p_{i}p_{i+1}$) values, which are established by neighboring points. Since human-body movements occur at joints, points with higher $\beta_{i}$ can be considered joints. Thus, the first step is to calculate $\beta_{i}$ formed by contiguous points $p_{i-1}$, $p_{i}$, and $p_{i+1}$. The calculation of $\beta_{i}$ uses the inner product formula. For $\beta_{1}$, ⋯, $\beta_{i}$, ⋯, $\beta_{n-1}$, points that correspond to substantial tilts are selected as joint candidates. Subsequently, $p_{k}$ and $p_{h}$ are identified by referencing $p_{i}$, which corresponds to the largest $\beta_{i}$.

Then, $l_{ak}$, $l_{kh}$, and $l_{hs}$ are calculated through distance computations involving $p_{a}$, $p_{k}$, $p_{h}$, and $p_{s}$. The values of $p_{k}$ and $p_{h}$ are sequentially assessed to determine the appropriateness of these estimations. If $l_{ak}$, $l_{kh}$, and $l_{hs}$ fall outside of the effective range of the lengths, alternative point candidates are calculated and substituted with new joint points. This calculation process is reiterated to identify $p_{a}$, $p_{k}$, $p_{h}$, and $p_{s}$, allowing for the computation of $\theta_{a}$, $\theta_{k}$, and $\theta_{h}$ using the inner product formula. This applies to a standing posture or a similar position. If all differences in $\beta_{i}$ are negligible, it will be challenging to ascertain $p_{k}$ and $p_{h}$. To mitigate this uncertainty, the length ratios of $l_{ak}$, $l_{kh}$, and $l_{hs}$ are empirically established as 0.35, 0.35, and 0.3, respectively, relative to $dist(p_{a},p_{s})$. These values are derived from research findings pertaining to Japanese body size [39].

### 3.5. SPD Module

The SPD module is illustrated using Figure 1 and Figure 2. The motions of standing up and sitting down are broadly categorized into two states: dynamic and static. The dynamic state includes the actions involved in transitioning to a standing or sitting position. First, the dynamic state of the standing-up motion is split into two phases: the forward flexion phase and the upward movement with the body extension phase. For ease of reference, these phases are labeled standing phase I ($S_{a}$-I) and standing phase II ($S_{a}$-II), respectively (Figure 1). Next, the dynamic state of the sitting-down motion is further divided into two phases, specifically, the downward movement associated with the sitting phase and the back extension phase, referred to as sitting phase I ($S_{i}$-I) and sitting phase II ($S_{i}$-II), respectively (see Figure 2). In contrast, the static state, encompassing both the standing and sitting positions, exhibits no variations in $\theta_{a}$, $\theta_{k}$, and $\theta_{h}$. Since these positions can also be viewed as distinct phases in the context of continuous movements, the standing position is designated as standing phase III ($S_{a}$-III), while the sitting position is denoted as sitting phase III ($S_{i}$-III).

**Figure 8:**
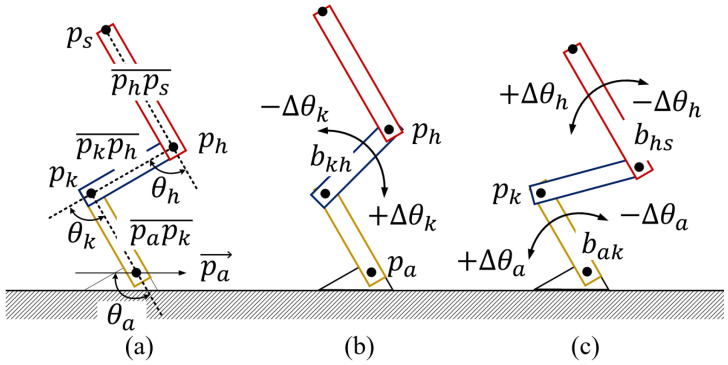
Pose explanation based on angle variations $\Delta \theta_{a}$, $\Delta \theta_{k}$, and $\Delta \theta_{h}$ of individual joint angles $\theta_{a}$, $\theta_{k}$, and $\theta_{h}$ ((**a**) definitions of $\theta_{a}$, $\theta_{k}$, and $\theta_{h}$, (**b**) $\Delta \theta_{k}$ variations, (**c**) $\theta_{a}$ and $\theta_{h}$ variations).

Here, we explain the relationships between the postures and the angles $\theta_{a}$, $\theta_{k}$, and $\theta_{h}$. The changes in these angles between time points t−1 and *t* are denoted as $\Delta \theta_{a}$, $\Delta \theta_{k}$, and $\Delta \theta_{h}$, respectively. As shown in Figure 8, when $\Delta \theta_{a}$ is positive, $b_{ak}$ bends forward while the knee is pushed outward; conversely, when $\Delta \theta_{a}$ is negative, $b_{ak}$ extends. Furthermore, if $\Delta \theta_{k}>0$, both $b_{ak}$ and $b_{kh}$ at $p_{k}$ bend, causing $p_{h}$ to move backward. In contrast, when $\Delta \theta_{k}<0$, $p_{h}$ is lifted forward owing to an extension movement. Additionally, when $\Delta \theta_{h}$ is positive, $b_{kh}$ and $b_{hs}$ at $p_{h}$ bend forward, whereas when $\Delta \theta_{h}$ is negative, $b_{kh}$ and $b_{hs}$ at $p_{h}$ extend.

Considering these conditions and assumptions, we propose a phase determination algorithm (Algorithm 1) for the SPD module. The inputs for Algorithm 1 include $l_{ts}\left(t\right)$ from the distance estimation module and $\theta_{a}$, $\theta_{k}$, and $\theta_{h}$ from the joint angle estimation module. The phase determination computation uses these inputs, producing one of six possible phases at *t*.
**Algorithm 1:** Standing/sitting phase determination.
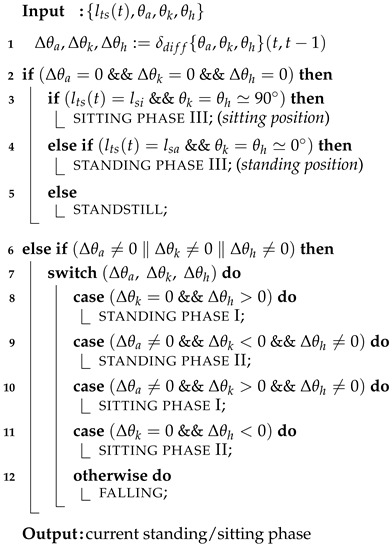


The details of the computation are as follows. Figure 1a,d and Figure 2a,d illustrate the static state, where $\Delta \theta_{a}$, $\Delta \theta_{k}$, and $\Delta \theta_{h}$ are all 0 (indicating that $\theta_{a}$, $\theta_{k}$, and $\theta_{h}$ are stationary). As noted in Section 1, an individual may pause while either standing up or sitting down. To avoid incorrect phase determination, $l_{ts}\left(t\right)$ is used to verify if the current phase is $S_{a}$-III or $S_{i}$-III. When $\theta_{k}=\theta_{h}≃0^{{}^{\circ}}$ and $l_{ts}=l_{sa}$, this indicates a standing position as shown in Figure 1d and Figure 2a. Conversely, when $\theta_{k}=\theta_{h}≃90^{{}^{\circ}}$ and $l_{ts}=l_{si}$, it indicates a sitting position as depicted in Figure 1a and Figure 2d.

Next, each phase is identified based on the variations in $\theta_{a}$, $\theta_{k}$, and $\theta_{h}$. As depicted in Figure 1b, in $S_{a}$-I, the body bends forward while the knee remains fixed. This phase is recognized when $\Delta \theta_{k}=0$ and $\Delta \theta_{h}>0$. $S_{a}$-II, shown in Figure 1c, commences when the buttocks leave the stool’s surface and concludes just before the individual reaches a standing position. During this phase, the center of gravity of the body rises, and $\theta_{k}$ is extended. $S_{a}$-II is identified when $\Delta \theta_{a}\neq 0$, $\Delta \theta_{k}<0$, and $\Delta \theta_{h}\neq 0$. As seen in Figure 2c, $S_{i}$-I begins at the standing position and ends when the buttocks reconnect with the stool surface. During this phase, the body posture shifts downward through the bending of joint angles. Consequently, $S_{i}$-I is identified when $\Delta \theta_{a}\neq 0$, $\Delta \theta_{k}>0$. As illustrated in Figure 2c, $S_{i}$-II encompasses the motion process until a seated position is achieved after the buttocks make contact with the seating surface. In this phase, the bent body rises. If $\Delta \theta_{k}=0$ and $\Delta \theta_{h}<0$, then $S_{i}$-II is determined. Finally, for any posture not included in the six previously mentioned processes, it is assumed that the individual is in a state of falling.

## 4. Evaluation Results and Discussion

### 4.1. Evaluation Direction and Experimental Settings

Five types of experiments were performed for the standing-up and sitting-down motions on two types of voluntary participants, namely, laboratory colleagues and anonymous individuals (including elderly people), to verify the effectiveness of the proposed representation scheme. Specifically, we examined the characteristics of the scheme using a proximity sensor and validated its performance by comparing measurement sensors. Before performing the experiments, we outlined the objectives of this study along with the experimental processes and settings to the participants. Subsequently, we obtained written informed consent from the voluntary subjects for their participation and for any accompanying images. Lastly, we obtained approval from the ethics committee of the University of Miyazaki for the experiments (approval No. 2021-002). Additionally, all experiments were performed in accordance with the protocol established by the University of Miyazaki Ethics Committee.

**Figure 9:**
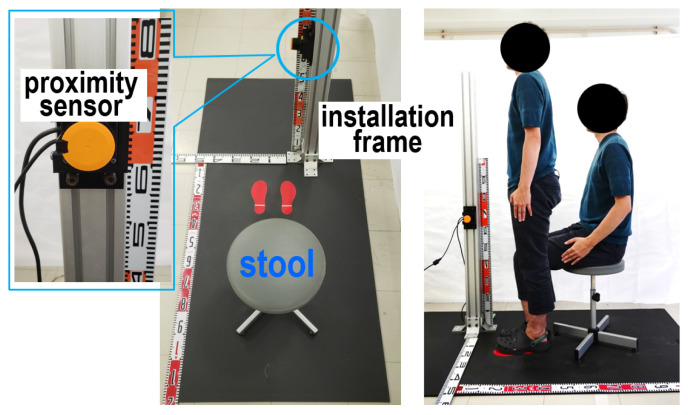
Experimental setting using proximity sensor (**left**) and experimental scene (**right**).

**Figure 10:**
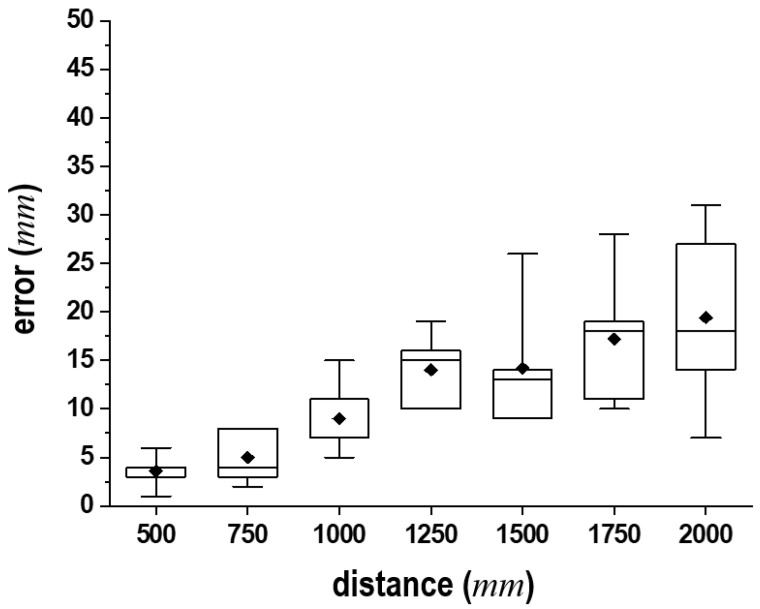
Experimental results for distance measurements at 250 mm intervals using the UST-10LX proximity sensor.

As illustrated in Figure 9, the experimental setup comprised a height-adjustable stool capable of adjusting $l_{c}$, a UST-10LX proximity sensor (Hokuyo Automatic Co., Ltd., Tokyo, Japan), and its supporting frame. Figure 10 shows the statistical analysis results from 100 trials, measuring distances at 250 mm intervals in a range of 500 mm to 2000 mm. Despite a slight decrease in the measurement accuracy, the proximity sensor demonstrated satisfactory performance for practical experimental applications. Subsequently, $l_{c}$ was adjusted to ensure that the subjects’ heels naturally made contact with the ground when seated. The subjects were instructed to wear lightweight clothing or to adjust their shirts and pants to minimize the distance from *S* to their bodies. The surfaces were measured. During these experiments, participants assumed the following postures: they were instructed to either cross their arms in front of their chest or let their arms rest by their sides. At times, the positioning of the hands interfered with the distances to the body surface, leading to inaccuracies in the distance measurements. These complications made it challenging to accurately detect the joint locations, resulting in some deviated data. Additionally, we asked participants to perform these motions slowly according to our command signals for standing up and sitting down to avoid causing them any strain. Each individual’s body was oriented toward the installation frame of *S* to facilitate distance measurements along the sagittal plane.

A FDR-AX700 digital 4K video camera recorder (SONY Corporation, Tokyo, Japan) and a Vantage V8 motion capture device (Vicon) (Inter Reha Co., Ltd., Tokyo, Japan) were used to evaluate the results of the proposed scheme. The standing-up and sitting-down motions were recorded on the $\vec{x}\vec{y}$ plane using the video camera, and the results were analyzed through video analysis for comparison. Additionally, the motion capture was performed to assess the results of the proposed scheme further. Furthermore, an EAS linear actuator (Orientalmotor Co., Ltd., Tokyo, Japan), simulating the standing-up or sitting-down motion, was used as an index for evaluating the phase determination.

### 4.2. Experimental Results in Laboratory Environment

**Figure 11:**
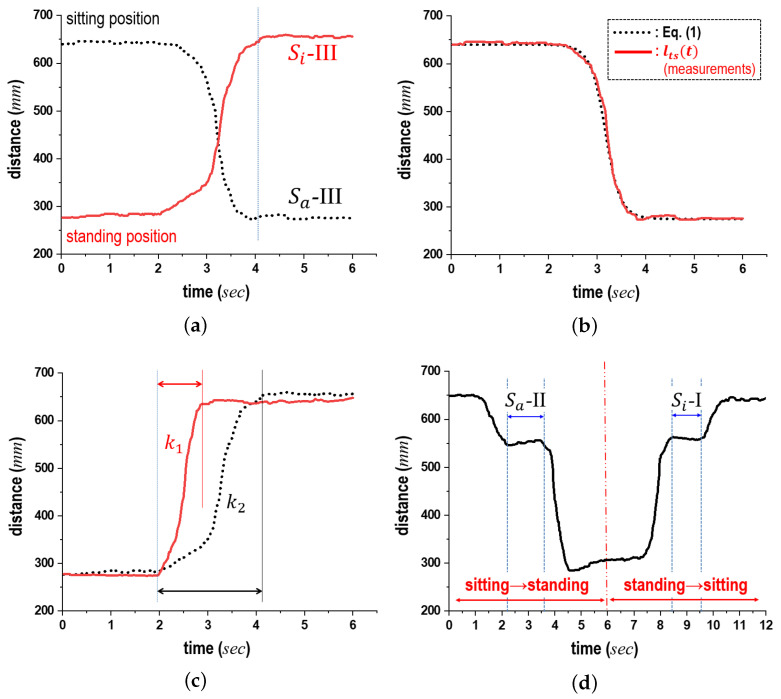
Experimental results obtained using distance estimation module. (**a**) Trajectories of $l_{ts}\left(t\right)$ when standing up and sitting down; (**b**) Comparative results between Equation (1) and $l_{ts}\left(t\right)$ (measurements); (**c**) Comparisons according sitting speeds $k_{1}$ and $k_{2}$; (**d**) Trajectories of $l_{ts}\left(t\right)$ when pausing in $s_{a}$-II and $s_{i}$-I.

The initial experiments were performed to validate the functionality of the distance estimation module, with the results presented in Figure 11. In this figure, the horizontal axis represents time (in seconds), and the vertical axis represents distance (in millimeters). The trajectories depicting the average $l_{ts}\left(t\right)$ values, measured over 10 trials while the subjects were performing standing-up and sitting-down motions, are shown in Figure 11a. The black dotted line represents the $l_{ts}\left(t\right)$ trajectories for the standing-up motion, while the red solid line indicates the trajectories for the sitting-down motion, respectively. These results confirmed that $l_{ts}\left(t\right)$ obtained during these motions was similar to the findings of Equation (1) (Figure 7a). Therefore, this module could adequately determine the standing and sitting positions and compute $l_{ts}\left(t\right)$ according to the standing-up and sitting-down motions.

In Figure 11b, the black dotted line represents Equation (1), while the red bold line corresponds to the measured $l_{ts}\left(t\right)$. As illustrated in Figure 7, Equation (1) and $l_{ts}\left(t\right)$ show a relatively close resemblance. Figure 11c shows that the speed differences during the sitting-down motion were evident between 2 and 4 s after the start. The black dotted line indicates the results of slow sitting-down motions, whereas the red solid line represents the results of normal sitting-down motions. As expected, a shorter motion time led to a steeper incline in relation to the change in distance. Whether standing up or sitting down quickly or slowly, variations in completion time are attributed to differences in motion speeds. It was concluded that the distance estimation module, by adjusting $k_{i}$ in Equation (1), is capable of monitoring both standing-up and sitting-down motions.

Figure 11d shows the trajectories of $l_{ts}\left(t\right)$ based on the following motion patterns. During the standing-up process, each subject was instructed to stop moving. After approximately 1 s, they were prompted to resume standing up. Subsequently, participants were asked to sit down from the standing position. In the middle of these movements, subjects were again instructed to stop. After approximately 1 s, they continued the downward motion. During these pauses, the module mistakenly classified the stop as a single phase, assigning the pausing commands to $S_{a}$-II and $S_{i}$-I. As detailed in Section 3.3, Equation (1) only identifies the static phase based on $l_{ts}\left(t\right)$. While elderly individuals may pause during transitions, these transitional states should be accurately represented.

**Figure 12:**
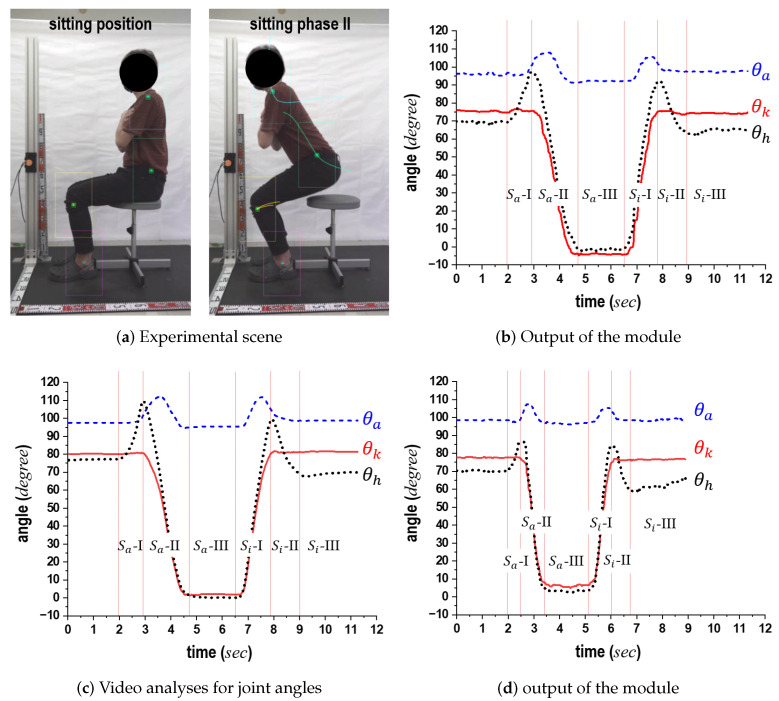
Computation and video analysis results for $\theta_{a}$, $\theta_{k}$, and $\theta_{h}$ are provided to evaluate the joint angle estimation module, with cases (**b**,**c**) representing trajectories obtained by the joint-angle estimation module and those obtained from the video clips, respectively, and with cases (**b**,**d**) representing the results for normal motions and swifter motions, respectively.

Second, to assess the effectiveness of the joint angle estimation module, we compared the module’s outputs with the image analysis results derived from video clips. Figure 12a shows the experimental setup. Markers were affixed to the ankle, knee, and hip of each participant. Their standing-up and sitting-down motions were simultaneously recorded by a video camera, allowing for the computation of $\theta_{a}$, $\theta_{k}$, and $\theta_{h}$ through the module. The trajectories of the three markers were extracted from the video clip.

Figure 12b shows the $\theta_{a}$, $\theta_{k}$, and $\theta_{h}$ trajectories calculated by the joint angle estimation module. Figure 12c presents the $\theta_{a}$, $\theta_{k}$, and $\theta_{h}$ trajectories extracted from the video clips recorded during the standing-up and sitting-down motions depicted in Figure 12b. In these figures, the horizontal axis represents time (in seconds), while the vertical axis indicates the angle (in degrees). The red vertical lines denote the boundaries of individual phases. The transition between the preparation phase and $S_{a}$-I (or $S_{i}$-I) corresponds to the moment when command signals were issued. Other boundary lines were marked based on the sitting position on the stool. Overall, the trajectories of $\theta_{a}$, $\theta_{k}$, and $\theta_{h}$ exhibited similar trends. Despite the small differences in the $\theta_{a}$, $\theta_{k}$, and $\theta_{h}$ values obtained from the module and those from the video clips, our comparisons demonstrated that the proposed module could calculate the individual angles based on only $d_{i}$ as explained in Section 3.2.

In a different experimental setting, unlike the scenarios depicted in Figure 12b,c, we instructed the subject to perform standing-up and sitting-down motions at a faster pace. Figure 12d shows the $\theta_{a}$, $\theta_{k}$, and $\theta_{h}$ trajectories for these motions. The results indicate that the overall characteristics of these trajectories displayed similar patterns. Owing to the increased speed of the actions in these experiments, the maximum $\theta_{h}$ observed was lower compared to those in Figure 12b. Notably, distinct speeds were reflected in $\theta_{h}$ during the standing-up and sitting-down motions. As a result, although the motions were performed 1.3 times faster when comparing the motion duration in Figure 12b with those in Figure 12d, the joint angle estimation module effectively monitored and calculated the variations in $\theta_{a}$, $\theta_{k}$, and $\theta_{h}$.

**Figure 13:**
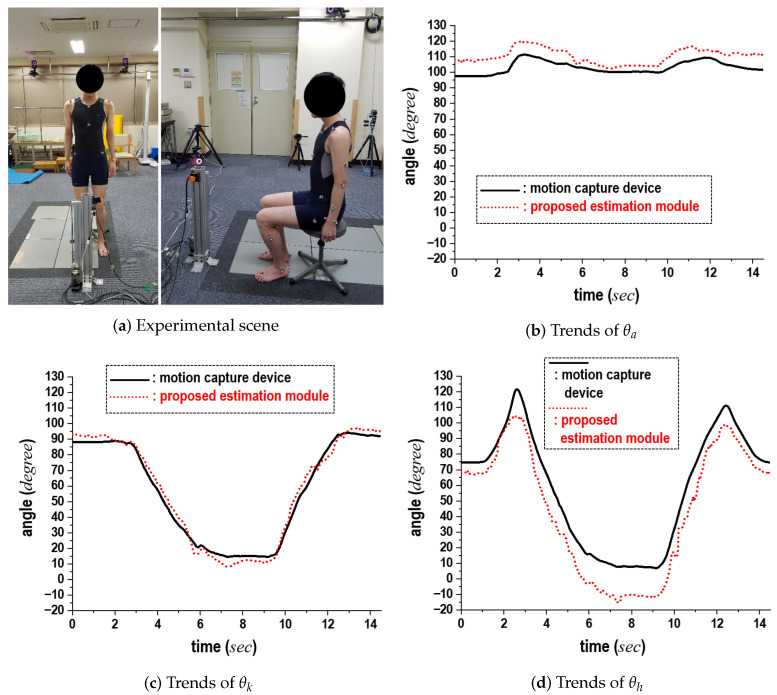
Comparison results between the joint-angle estimation module and the motion capture device.

The third experiment was performed to assess the feasibility of the joint angle estimation module by comparing its measurements to those from the motion capture device. Figure 13a shows the experimental setup with a young subject, while the results in Figure 13b–d show the $\theta_{a}$, $\theta_{k}$, and $\theta_{h}$ trajectories, respectively. In these graphs, the black solid line represents the motion capture results, and the red dotted line indicates the computation results from the proposed module (obtained using the proximity sensor). The horizontal and vertical axes represent time (in seconds) and angle (in degrees), respectively. The two sets of results showed a close approximation regarding both the time-change zones and angle magnitudes. Fluctuations in $\theta_{h}$ were noticeable, even though the trends of the $\theta_{a}$ and $\theta_{k}$ trajectories were comparable. Additionally, when compared to the results from the motion capture device, $\theta_{h}$ displayed the largest error, with an average deviation of up to $13^{{}^{\circ}}$. This increased error in $\theta_{h}$ occurred since, when flexed, the waist position was relatively distant from *S*, located in the front direction, making its measurement more challenging compared to $\theta_{a}$ and $\theta_{k}$. Despite this slight difference, we confirmed that the joint angle estimation module is capable of producing patterns similar to those measured by the motion capture.

**Figure 14:**
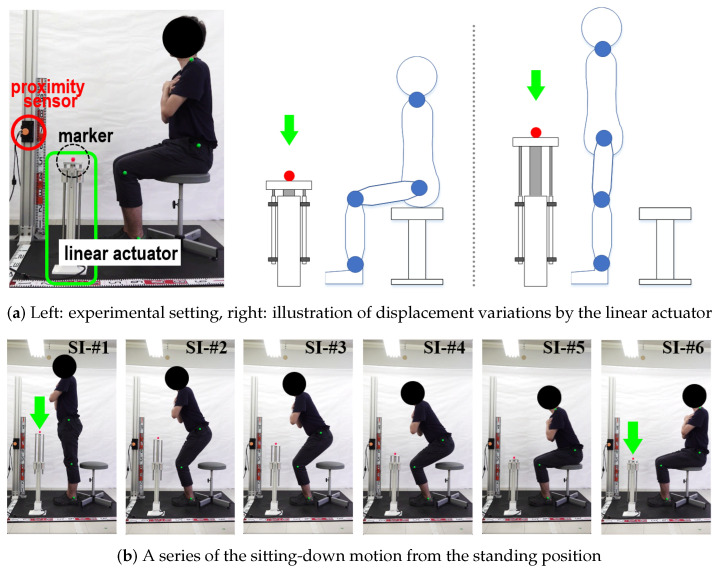
Experimental setup using linear actuator to evaluate the effectiveness of the SPD module.

Fourth, as detailed in Section 3.5, the SPD module (Algorithm 1) produced outputs for each motion phase at *t*. The effectiveness of this module was evaluated through experiments performed with a linear actuator. Figure 14a shows the experimental setup and provides a conceptual depiction of the experiments involving the linear actuator. This actuator was governed by the motions of each subject. During the standing-up (sitting-down) motions, the transitional displacements controlled by the linear actuator were specified as changes in the $l_{kh}$ projected onto $\vec{y}$ exclusively at $S_{a}$-II (and $S_{i}$-I), meaning the displacements of the subject’s $p_{h}$ in the $\vec{y}$ direction. In summary, the goal of this experiment was to assess the accuracy of the $\theta_{a}$, $\theta_{k}$, and $\theta_{h}$ values generated by the joint angle estimation module and to evaluate the reliability of the SPD module’s output. Under identical experimental conditions, as shown in Figure 12, we recorded the transitional movements using a camera, and the video clips were analyzed to simultaneously verify the subjects’ motions alongside those of the actuator. Figure 14b shows photographs of a subject executing the sitting-down motion from a standing position.

**Figure 15:**
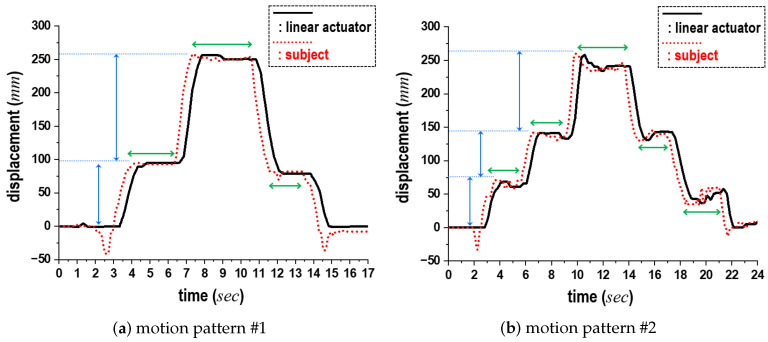
Comparison of displacements of linear actuator, shown in Figure 14, according to two patterns of subject movements.

As comparative results, Figure 15 shows the variations in the $l_{kh}$ projected onto $\vec{y}$, alongside the corresponding displacements of the linear actuator in the same direction when $S_{a}$-II (and $S_{i}$-I) are applied. In this figure, the black solid lines represent the displacements of the linear actuator, while the red dotted lines denote the $l_{kh}$ of the subject. The horizontal and vertical axes depict time (in seconds) and displacement (in millimeters), respectively. Additionally, the blue and green bidirectional arrows indicate the range of displacement observed during continuous movements and the pause intervals in these motions, respectively. (See video-S1: *Experimental-scene-1.mp4* submitted as Supplementary Materials).

**Figure 16:**
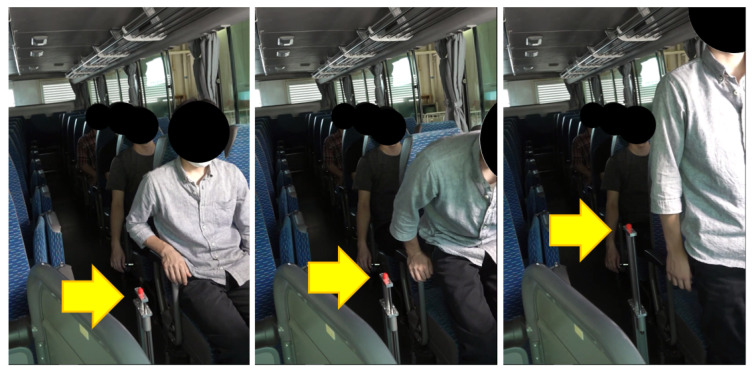
Experimental scene for our application example in a bus.

As illustrated in Figure 15a, each subject paused once during both the standing-up and sitting-down motions (motion pattern #1). Despite these pauses, the linear actuator rose and subsequently returned to its initial position. In contrast, during the second trial shown in Figure 15b, we instructed each subject to pause twice during the standing-up and sitting-down motions (motion pattern #2). This required the subjects to maintain unfamiliar pause postures as directed, leading to body swaying. Consequently, minor fluctuations were noted during the preparation phase, as the subjects readied themselves for their next movements or during the pauses. Upon analysis of the data, the average displacement of these fluctuations was recorded at 3 cm before the initiation of the motions and 5 cm during the paused postures. We initially attributed the delay in the actuator’s motions, which was approximately 0.3 s, to the acceleration and deceleration processes. From a hardware perspective, we observed that the standing-up and sitting-down motions coincided with similar displacements of the actuator, which confirmed the accuracy of the SPD module. Finally, Figure 16 presents the experimental results from the feasibility test of one target application, wherein the proposed scheme monitors the standing-up and sitting-down actions of a passenger in a bus. According to these motions, the marker of the linear actuator ascends and descends, respectively.

### 4.3. Experimental Results for Elderly and Voluntary Subjects

We performed experiments to assess the usability of the proposed representation scheme for anonymous subjects with individual differences. The participants included 58 male and female volunteers: 4 in their 20 s, 5 in their 30 s, 8 in their 40 s, 11 in their 50 s, 11 in their 60 s, 14 in their 70 s, and 5 in their 80 s. Approximately 33% of the participants were elderly (aged 70 and above). As illustrated in Figure 14, comparative experiments were performed at a nursing home and during a regional welfare event, with the results from the scheme being thoroughly analyzed. (See video-S2: *Experimental-scene-2.mp4* and video-S3: *Experimental-scene-3.mp4* submitted as Supplementary Materials).

**Figure 17:**
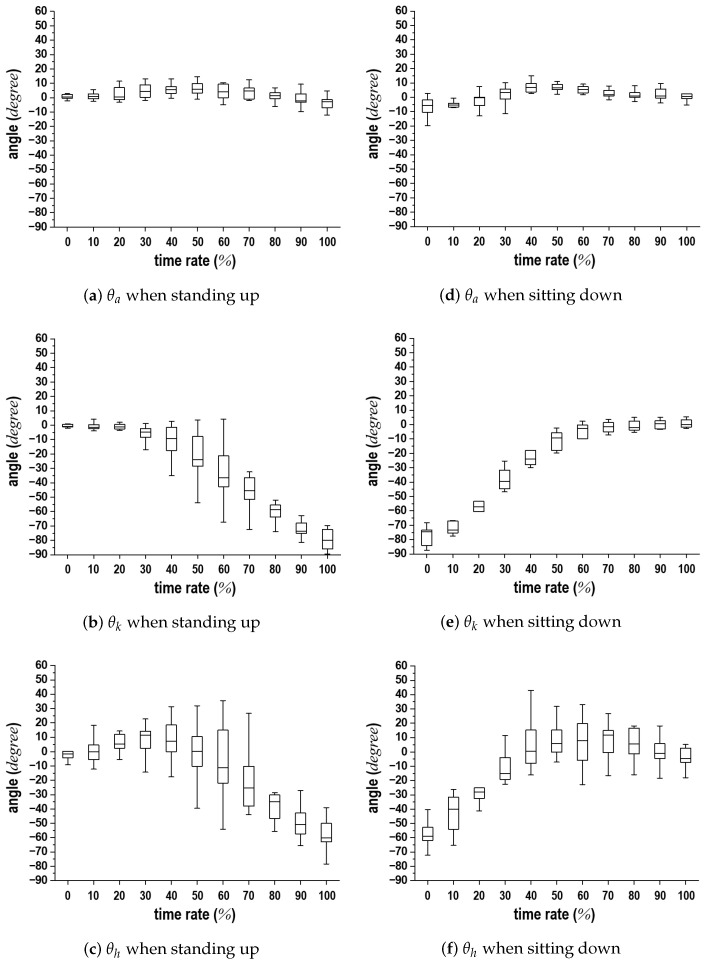
Statistical trends of experimental results for $\theta_{a}$, $\theta_{k}$, and $\theta_{h}$ over time rate (%) during standing-up and sitting-down motions of 58 subjects.

The statistical data from the experiments are shown in Figure 17. In this figure, the boxes represent the confidence intervals of 25–75%, the horizontal bars in the boxes indicate the mean values, and the error bars denote the standard deviations. Figure 17a,d, Figure 17b,e, and Figure 17c,f depict the statistical variations in $\theta_{a}$, $\theta_{k}$, and $\theta_{h}$ over time taken for the standing-up and sitting-down motions. Each subject exhibited unique motion patterns and speeds during these movements, making it challenging to analyze the data of all participants without an appropriate standard. Consequently, we developed a comparison standard based on individual variations. The joint angle in the sitting position was designated as the initial value of $0^{{}^{\circ}}$; thus, the comparison standard was defined as the change in angle over the duration needed to complete the standing-up or sitting-down motion.

The trends observed in Figure 17 were consistent with those in Figure 12 and Figure 13, despite the variations in motion speeds attributed to individual differences. The median values indicate that the displacement ranges for each joint during these motions were between $7^{{}^{\circ}}$ and $-7^{{}^{\circ}}$ relative to the initial $\theta_{a}$, between $0^{{}^{\circ}}$ and $-78^{{}^{\circ}}$ relative to the initial $\theta_{k}$, and between $10^{{}^{\circ}}$ and $-58^{{}^{\circ}}$ relative to the initial $\theta_{h}$. As demonstrated in the submitted video clip, individual differences in the standing-up and sitting-down motions were influenced by personal habits, muscle strength, and body types. It is believed that this contributed to the increase in the error bar at $\theta_{k}$ and $\theta_{h}$. These ranges can serve as an index for the variability in the joint angles. As a result, different motion trends can be represented despite individual differences leading to variations in motion speeds. Furthermore, the proposed distance-based representation scheme successfully estimated $\theta_{a}$, $\theta_{k}$, and $\theta_{h}$, confirming that the six phases could be identified even among subjects with differing motion speeds.

### 4.4. Discussion

The findings from the experiments mentioned above are summarized as follows. First, the $d_{i}$ measurement and the estimation of $\theta_{a}$, $\theta_{k}$, and $\theta_{h}$ using the geometric model showed trends in their angle transitions that closely aligned with the joint angles obtained through motion capture. This confirms the efficacy of the angle estimation method. Second, to assess the effectiveness of the SPD module, we validated the phase determination and the displacements of $l_{kh}$ derived from $\theta_{a}$, $\theta_{k}$, and $\theta_{h}$ during standing-up and sitting-down motions through comparative experiments involving a linear actuator and multiple subjects. Third, we performed experiments on 58 voluntary subjects and found that the proposed scheme could be applied to cases involving individual differences during the standing-up and sitting-down motions.

These analyses suggest that this study is valuable for interface development. However, dispersions were observed in the transition processes for $\theta_{a}$, $\theta_{k}$, and $\theta_{h}$ due to several factors. The proximity sensor measurements relied on the distances from the sensor to the body surface, while the video camera and motion capture device tracked markers attached to the body. Additionally, the type of clothing worn also contributed to these variations. The upper garments worn by the subjects were specifically designed for the experiments; however, the lower garments were not designated for this purpose. Some hems were wide and shifted during movement. For the postures in these experiments, subjects were asked to either cross their arms in front of their chests or place their arms at their sides. Given the intended applications, it is undesirable to impose such restrictions on the positions and locations of the hands. One of our ongoing projects focuses on the accuracy of the measurement setups in relation to these factors. Additionally, we are developing an algorithm for situations where only a portion of the body is visible owing to obstacles that restrict the sensor’s line of sight.

This study will have a considerable impact on the field of assistive systems for lower limbs because the proposed scheme prioritizes user privacy and can seamlessly integrate into daily routines. However, there is still potential for improvement. A measurement method capable of working with thick clothing, skirts, or textured fabrics will be necessary to implement this representation scheme in everyday life. Furthermore, the proposed measurement method uses a proximity sensor to scan the body surface from the shoulder to the toes on the sagittal plane. As such, during experiments, subjects were instructed to stand in easily scannable positions. Taking these factors into account, future works can further refine the measurement method, including sensor installation, to develop an interface system based on this scheme.

## 5. Conclusions

An interface scheme that uses relative distance measurements to represent the motions of standing up and sitting down is introduced. This scheme encompasses the measurement of relative distance from a proximity sensor to the body surface, data reduction to a 2D coordinate plane, and the determination of motion phases based on variations in joint angles. Additionally, the transition process of the joint locations is captured by modeling the human body as a geometric linkage structure. Using a proximity sensor, the recognition of standing-up and sitting-down motions requires less data compared to other measurement methods, such as cameras. The sensors do not need to be affixed to the body for measurement purposes. Furthermore, individual privacy is protected since the scheme does not require extensive data that could identify individuals. Comparative experiments were performed with subjects of varying ages to showcase the effectiveness and performance of the proposed approach. While minor fluctuations in the experimental results were noted owing to individual variations among participants, the proposed scheme proved to be satisfactorily effective. In conclusion, the scheme emphasizes the following key aspects: noncontact handling in the post-COVID-19 era and the recognition of the discontinuous transition process between standing and sitting positions, and vice versa. The proposed scheme is applicable to the interfaces of welfare and measurement devices in medical and welfare facilities, among others. Our future research will focus on improving the measurement accuracy of the developed method and exploring its applications in public spaces.

## Data Availability

Data are contained within the article.

## Supplementary Materials

The following supporting information can be downloaded at: https://www.mdpi.com/article/10.3390/s24216967/s1. The first video clip shows the experimental results obtained with the linear actuator on one laboratory colleague to examine the effectiveness of the SPD module as shown in Figure 14 and Figure 15. The second video clip shows the experimental scene in the nursing home. The results are shown in Figure 17. The third video clip presents the experimental scene with the voluntary subjects at the regional event. The related results are shown in Figure 17.

## Abbreviations

The following abbreviations are used in this manuscript:

1D	one-dimensional
2D	two-dimensional
SPD	standing/sitting phase determination
